# Time spent with cats is never wasted: Lessons learned from feline acromegalic cardiomyopathy, a naturally occurring animal model of the human disease

**DOI:** 10.1371/journal.pone.0194342

**Published:** 2018-03-29

**Authors:** Kieran Borgeat, Stijn J. M. Niessen, Lois Wilkie, Norelene Harrington, David B. Church, Virginia Luis Fuentes, David J. Connolly

**Affiliations:** 1 Clinical Science and Services, Royal Veterinary College, Hatfield, United Kingdom; 2 Langford Veterinary Services, University of Bristol, Bristol, United Kingdom; 3 Pathology and Pathogen Biology, Royal Veterinary College, Hatfield, United Kingdom; Scuola Superiore Sant'Anna, ITALY

## Abstract

**Background:**

In humans, acromegaly due to a pituitary somatotrophic adenoma is a recognized cause of increased left ventricular (LV) mass. Acromegalic cardiomyopathy is incompletely understood, and represents a major cause of morbidity and mortality. We describe the clinical, echocardiographic and histopathologic features of naturally occurring feline acromegalic cardiomyopathy, an emerging disease among domestic cats.

**Methods:**

Cats with confirmed hypersomatotropism (IGF-1>1000ng/ml and pituitary mass; n = 67) were prospectively recruited, as were two control groups: diabetics (IGF-1<800ng/ml; n = 24) and healthy cats without known endocrinopathy or cardiovascular disease (n = 16). Echocardiography was performed in all cases, including after hypersomatotropism treatment where applicable. Additionally, tissue samples from deceased cats with hypersomatotropism, hypertrophic cardiomyopathy and age-matched controls (n = 21 each) were collected and systematically histopathologically reviewed and compared.

**Results:**

By echocardiography, cats with hypersomatotropism had a greater maximum LV wall thickness (6.5mm, 4.1–10.1mm) than diabetic (5.9mm, 4.2–9.1mm; Mann Whitney, p<0.001) or control cats (5.2mm, 4.1–6.5mm; Mann Whitney, p<0.001). Left atrial diameter was also greater in cats with hypersomatotropism (16.6mm, 13.0–29.5mm) than in diabetic (15.4mm, 11.2–20.3mm; Mann Whitney, p<0.001) and control cats (14.0mm, 12.6–17.4mm; Mann Whitney, p<0.001). After hypophysectomy and normalization of IGF-1 concentration (n = 20), echocardiographic changes proved mostly reversible. As in humans, histopathology of the feline acromegalic heart was dominated by myocyte hypertrophy with interstitial fibrosis and minimal myofiber disarray.

**Conclusions:**

These results demonstrate cats could be considered a naturally occurring model of acromegalic cardiomyopathy, and as such help elucidate mechanisms driving cardiovascular remodeling in this disease.

## Introduction

Clinical and pathologic correlates between feline and human hypertrophic cardiomyopathy (HCM), arrhythmogenic right ventricular cardiomyopathy (ARVC) and restrictive cardiomyopathy (RCM) have been previously identified [1–4]. As a result, these feline cardiomyopathies are believed to represent naturally occurring animal models of the equivalent human conditions. The influence of a number of systemic diseases on the human myocardium have been thoroughly documented but such changes are less well characterised in cats, other than the hypertrophic effect of hyperthyroidism [5–14].

Hypersomatotropism (HS), resulting from a functional pituitary adenoma or hyperplasia of the *pars distalis* of the anterior pituitary gland, causes a syndrome of growth hormone (GH) excess: in skeletally mature patients, this results in the disease known as acromegaly. In humans with HS, cardiovascular disease is a significant contributor to morbidity and mortality [15–17] with approximately 60% of patients suffering a cardiovascular cause of death [18]. Even without clinical evidence of heart failure, increases in left ventricular (LV) mass and echocardiographic measures of LV wall thickness are observed, in addition to impaired overall cardiac performance and a high prevalence of valve disease [19–23]. Furthermore, higher IGF-1 and GH concentrations are significantly associated with worse cardiac dysfunction and increased LV mass, independent of impaired glucose tolerance and systemic hypertension [24]. Histopathology typically detects myocardial fibrosis and myocyte hypertrophy, with some samples demonstrating cellular infiltrates or small vessel disease in addition [25].

Like humans, cats may develop HS in middle to old age and similar to humans, the disease is thought to be increasing in prevalence. Recent work indicates that 25–33% of diabetic cats have HS [26, 27] which suggests that the disease is significantly more common in cats than in humans. Cats with HS most frequently present with signs consistent with of diabetes mellitus (DM) [26–31] although case reports of non-diabetic acromegalic cats also exist [32, 33].

Recently, a study evaluating environmental toxin levels in plasma of cats with acromegaly has suggested a link between organohalogenated contaminants and the etiology of acromegaly, which has led to similar studies in human acromegalics [34].

Although the physical manifestations of acromegaly on tissue growth can be subtle in the early phases of the disease, they have been well characterised in cats [27, 28, 35, 36]. However, the cardiovascular effects of feline HS are less well described. To date, only two case series have been published reporting a high prevalence of cardiac disease and heart failure in acromegalic cats [28, 37]; however, the value of these reports is limited by small numbers and a lack of control groups with which to draw comparisons. An additional confounding factor in feline studies is the high prevalence of hypertrophic cardiomyopathy (HCM) estimated at approximately 15%, with a higher prevalence in aged individuals [38–40]. It is possible that a large proportion of the echocardiographic and histopathological findings previously reported in feline HS actually reflected primary incidental HCM (defined as a single diastolic measurement of left ventricular wall thickness ≥6mm) rather than myocardial changes directly caused by acromegaly. A further complication is the potential confounding effect of diabetes on the feline myocardium. Although diabetes causes LV hypertrophy in humans [41,42], the cardiovascular effect of diabetes in cats is poorly understood. One retrospective case series did report a greater prevalence of cardiac disease in diabetic cats than age-matched controls [43], whilst another study evaluating serial echocardiographic changes in cats with DM suggested that diastolic dysfunction was associated with the diabetic state [44]. No studies investigating the potential pro-hypertrophic effect of DM in cats have been published.

The objective of this study was to describe a specific cardiomyopathy in cats associated with spontaneously occurring HS, whilst accounting for potential confounding factors such as HCM and diabetic cardiomyopathy, and to explore how closely this mirrors the syndrome recognised in humans.

## Methods

### Patient selection

**Fig 1:**
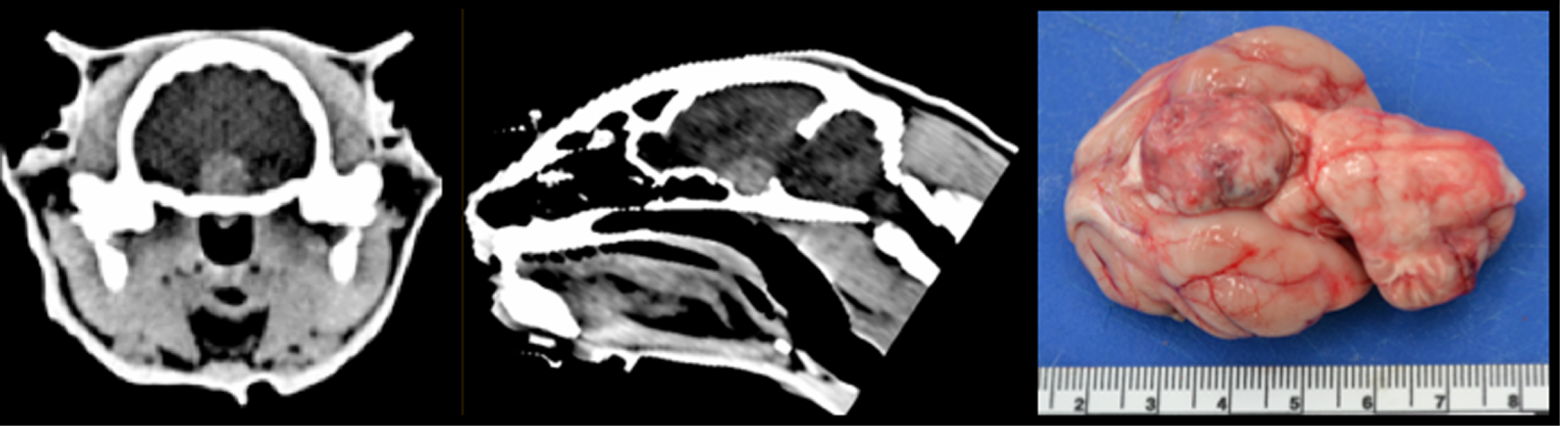
Composite image to demonstrate a pituitary mass typical of cats with acromegaly. Transverse (left) and saggital (middle) CT images showing a contrast enhancing pituitary mass (>4mm height) in a cat with acromegaly. Without treatment, the cat was euthanised some months later and post-mortem examination demonstrated the large pituitary mass (right). Histopathology was consistent with an acidophil adenoma.

This study did not involve the use of experimental animals. Ethical approval was granted by the Royal Veterinary College Ethical Review Board (*URN 2011 1120; URN 2017 1734–2*) and written owner consent for study participation was obtained. Healthy cats (controls) were recruited through a geriatric cat clinic organised in two veterinary general practices. Data on cats with diabetes mellitus but without acromegaly (DM group) and those with DM and HS (HS group) were prospectively collected between October 2009 and October 2016 at a veterinary teaching hospital. Inclusion criteria for the HS group were plasma IGF-1 >1000ng/ml and a contrast enhancing pituitary enlargement detectable on computed tomography (CT) imaging (pituitary height > 4.0mm; Fig 1) [26]. All HS cats were diabetic at the time of enrolment. Inclusion criteria for the DM group were clinical signs and clinicopathologic findings consistent with DM and a circulating IGF-1 concentration <800 ng/ml [29]. Diabetic cats with equivocal results for the diagnosis of HS (IGF-1 >800ng/ml but <1000 ng/ml, or >1000 ng/ml with CT imaging not performed or yielding equivocal findings) were excluded from the study. Inclusion criteria for the healthy control group were no evidence of systemic disease on physical examination and routine haematology and biochemistry analysis, no glycosuria on urinalysis and a circulating IGF-1 concentration <800ng/ml.

Exclusion criteria applicable to all groups were: previous diagnosis or treatment for cardiac disease, diagnosis of hyperthyroidism or other concurrent endocrinopathies, or inappropriate temperament for echocardiography or venepuncture. Cats with previously diagnosed systemic hypertension were also excluded, but cats presenting to the clinic with a measurement of high systolic blood pressure (>170mmHg) were still included if fundoscopy did not detect changes consistent with hypertensive retinopathy [45].

### Clinical procedures

All cats underwent systolic blood pressure measurement (Doppler technique)(45), echocardiography and venepuncture. Blood sampling was performed for clinical purposes and samples were taken before insulin administration and following 8–10 hours of food withdrawal. Residual serum and ethylenediaminetetraacetic acid (EDTA) plasma were separated into aliquots and stored at -80C within 30 minutes of collection.

Cats that were treated for HS had serial echocardiography and blood sampling performed. Treatment decisions were based upon owner choice and clinician advice at the time of diagnosis and alterations to insulin therapy were made according to individual requirements by or under the supervision of a Board certified veterinary Internist (SN). Options for treatment were medical, using long-acting once monthly somatostatin analogue pasireotide (SOM230; Signifor™, Novartis, Basel, Switzerland) injections (8 mg/kg) [46], or surgical through trans-sphenoidal hypophysectomy [47].

Echocardiography was performed by or under the supervision of a Board certified veterinary cardiologist, using standardised equipment (Vivid 7 ultrasound machine, 7s probe; GE Systems, Hatfield, Hertfordshire, UK) and protocol [1–4]. Studies were recorded from conscious, unsedated animals using a 7.5mHz phased array transducer. Measurements were an average of 3 consecutive cardiac cycles and were performed offline by a single operator (KB), blinded to patient group and timing of echocardiography. Data were entered into an electronic database, along with patient details (age, sex, breed, neuter status and study group).

Circulating concentrations of IGF-1, frustosamine and total T4 were measured from blood samples. Residual samples were stored and batch analysis of cardiac biomarkers (NTproBNP and cardiac troponin I) was performed after completion of the study by a commercial reference laboratory (IDEXX Laboratories Ltd, Wetherby, Yorkshire, UK).

### Histopathological investigation

For the purpose histopathological investigations, records of the Royal Veterinary College Anatomic Pathology Service tissue archive were searched for the following:

1. Geriatric cats (control group) without previously diagnosed heart disease and no clinical or necropsy evidence of DM or HS.
2. Cats with confirmed HCM which was associated with clinical signs of congestive heart failure or suspected sudden cardiac death (SCD) [48].
3. Cats with confirmed HS, irrespective of history of cardiac clinical signs.

Age-matching of cats in the HCM, control and HS groups was attempted. The possible inclusion of cats with occult HCM in the control group was an intentional aim to make the group more representative of a population of cats where HCM is commonplace in middle to old-age [40]. This allowed for more appropriate between-group comparisons, since it recognises the possibility that cats in the HS group may have had concurrent, primary HCM in addition to any potential secondary changes induced by their systemic endocrine disease.

A histopathological diagnosis of HS was made if acidophil hyperplasia, adenoma or adenocarcinoma of the *pars distalis* of the anterior pituitary at necropsy was confirmed by a Board-certified pathologist or a Resident under direct supervision. All reports were reviewed by a single Board-certified pathologist (NH), who also repeated the histopathological exam on any stored pituitary tissue which was available for review. In this study, hyperplastic lesions were characterised as multiple non-encapsulated well-demarcated small proliferations of acidophils that do not compress adjacent parenchyma. Acidophil adenomas were focal well-demarcated, expansile masses replacing and compressing normal parenchyma occasionally bordered by a thin partial to complete fibrous capsule [49]. Cases with concurrent disease processes which could contribute to LVH (evidence of adenomatous thyroid hyperplasia or thyroid carcinoma) [9, 50], and evidence of end-organ damage characteristic of systemic hypertension [51] were excluded. Clinical records were manually reviewed by a single operator (LW) for age, breed, sex, history of DM, IGF-1 concentration, echocardiographic findings (if present), treatment provided, and cause of death if known.

Gross pathology reports were reviewed for body weight (kg), heart weight (g) and thickness (mm) of the right ventricular free wall (RVFW), interventricular septum (IVS) and left ventricular free wall (LVFW) on a transverse cross section (perpendicular to the long axis of the ventricles) a third of distance from apex to base from epicardium to endocardium, excluding papillary muscles, as a single measurement using a millimetre rule; as previously described in cats [48]. The expected ratio of RVFW: IVS: LVFW is 1:3:3 and the reference range for relative heart weight used was 0.28–0.88% of body weight [52].

Cardiac histopathology slides of sections from cardiac tissue preserved in 4% neutral buffered formalin and routinely embedded in paraffin blocks were stained with hematoxylin-eosin and Masson trichrome. Slides were reviewed by a single feline cardiac pathologist (LW) in a blinded manner. Using a high-definition microscope (Olympus BX51TF, Olympus, Southend-on-Sea, UK), section(s) of myocardium on each slide were initially scanned at low power (x10 and x20 magnifications). Representative areas were subsequently examined systematically at x40 and x100 magnification, respectively.

Myocyte hypertrophy, myofiber disarray, interstitial, perivascular and replacement fibrosis and small intramural artery pathology were scored in a representative section, which was a transverse section to include LVFW and IVS wherever available, at x40 magnification as previously described [48]. In the absence of a published reference interval for feline myocyte size, scoring of hypertrophy was semiquantitative, and no quantitative analysis of myocyte size was made. Myocyte hypertrophy was defined as large myocytes with large, rectangular, hyper-chromic nuclei and graded as follows: 0, absent; 1, mild-focal; 2, moderate-multifocal; 3, severe-diffuse. Evaluation of myofiber disarray was performed in the left ventricle only. Disarray is common and normal in the right ventricle, owing to trabeculation, and also at the junctions between the free-walls and septum, within the dividing points of trabeculae, and around blood vessels. Assessment was made only on regions of longitudinally orientated myofibers. Fibrosis was classified as follows: interstitial, strands of connective tissue surrounding myocytes; replacement, confluent areas of fibrosis surrounding myocytes; perivascular, surrounding blood vessels; and subendocardial, fibrosis below and including the endocardium.

### Statistical analysis: Echocardiographic study

Data were assessed graphically for normality, which was tested using Shapiro-Wilk tests. Normally distributed data was represented mean (±standard deviation) and non-normally distributed data was represented median (range).

Categorical variables were compared between groups using Chi square/Fisher’s Exact tests. Normally distributed continuous variables were tested between the three groups using a one-way ANOVA, with pairwise comparisons using an independent samples t test. Non-normally distributed continuous variables were compared using a Kruskal-Wallis test, with pairwise comparisons using a Mann-Whitney U test. Significance was set at 5% with Bonferroni correction of p values for multiple comparisons.

To assess for a treatment effect, echocardiographic variables were compared before and after treatment with either pasireotide or hypophysectomy, using a Wilcoxon signed-rank test.

### Statistical analysis: Histopathological study

Histopathological variables were analysed to evaluate any significant differences between the three groups (controls, HCM and HS cats). Normality was assessed as before. Myocyte hypertrophy and myofibre disarray were converted to dichotomous variables (present/absent) for statistical analysis. These and other categorical variables were analysed using a Chi squared/Fisher’s Exact test. Age, heart weight, heart weight: body weight ratio, RVFW, IVS and LVFW thickness were compared between groups using a Kruskal-Wallis test and body weight was compared using a one-way ANOVA. Post-hoc pairwise comparisons and Bonferroni adjustment were performed on significant results. P values <0.05 were considered statistically significant overall.

## Results

### Echocardiographic differences at enrolment

**Fig 2:**
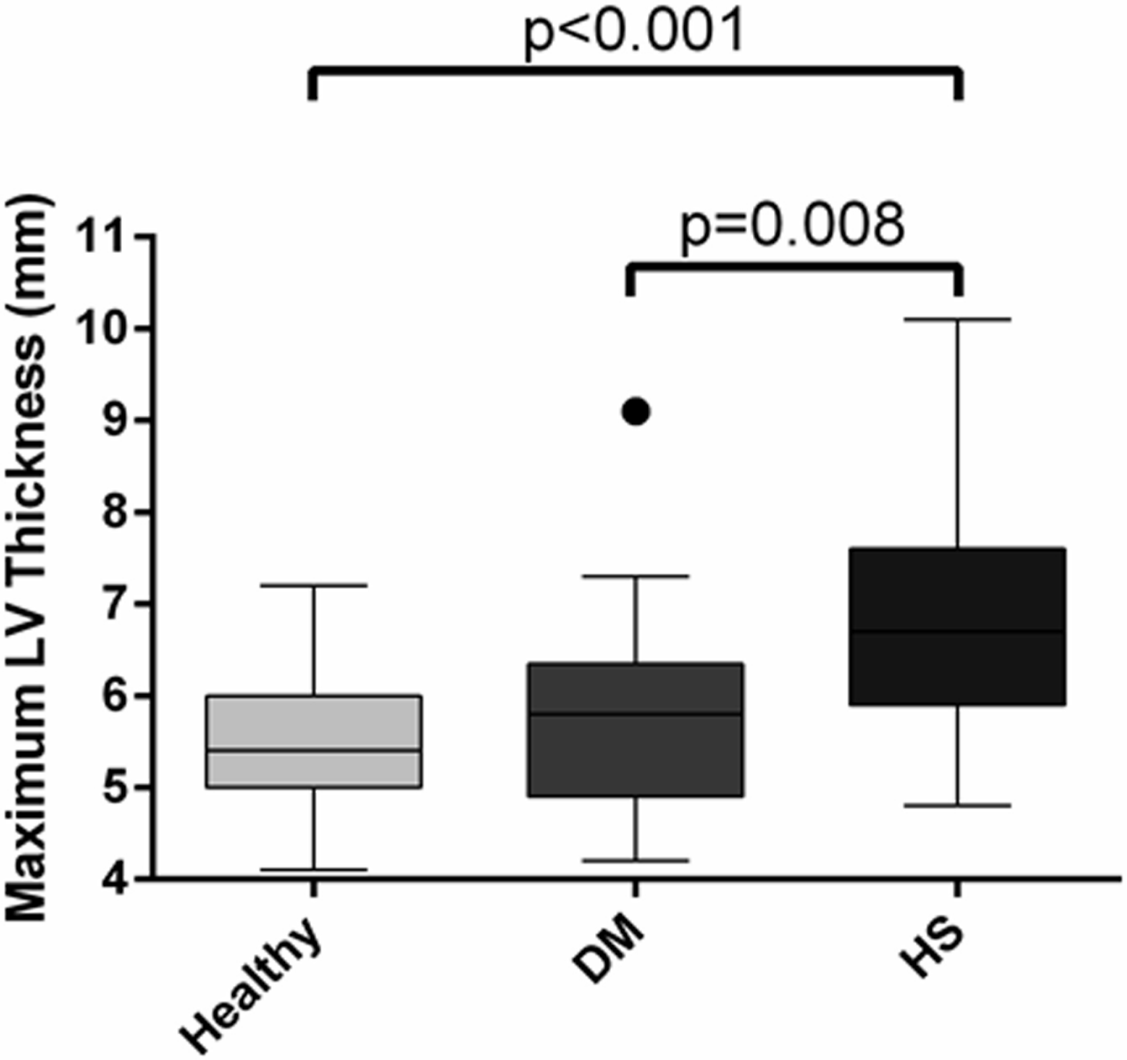
Box and whisker plot to illustrate the difference in maximum left ventricular (LV) wall thickness between the three groups of cats at enrolment. DM, diabetes mellitus; HS, hypersomatotropism.

**Fig 3:**
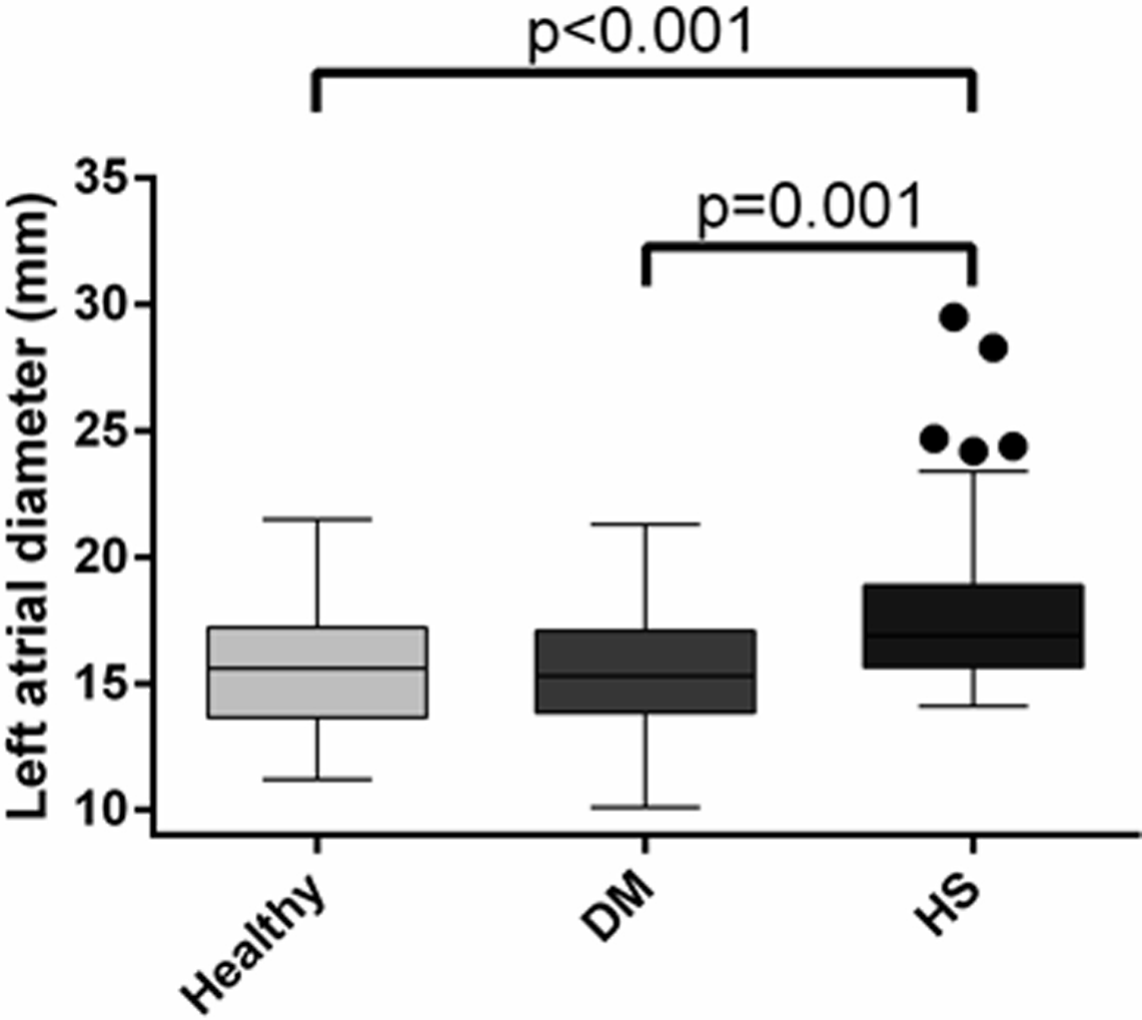
Box and whisker plots to illustrate the difference in left atrial size between groups at enrolment. DM, diabetes mellitus; HS, hypersomatotropism.

A total of 16 cats were enrolled in the control group, 24 cats in the DM group and 67 cats in the HS group. There was no significant difference in the baseline population characteristics of the three groups, other than HS cats being younger and heavier than cats in the other two groups (Table 1). All cats of both sexes had been neutered years prior to study enrolment. Maximum LV wall thickness was significantly greater in cats with HS (6.5mm, 4.4–10.1mm) than DM (5.9mm, 4.2–9.1mm; p = 0.008) or healthy cats (5.2mm, 4.1–6.5mm; p<0.001; Fig 2). Clinically significant LV hypertrophy (wall thickness ≥6mm) was present in 72% of the HS group, compared to 36% of diabetic cats and 21% of controls. Left atrial diameter was greater in the HS group (16.6mm, 13.0–29.5mm) than both cats with DM (15.2mm, 10.2–21.3mm; p = 0.001) and controls (14.0mm, 12.6–17.4mm; p<0.001, Fig 3). Aortic insufficiency was more common in both HS and DM cats than in healthy controls (p<0.001 and p = 0.018 respectively). A greater proportion of cats with HS exhibited reduced diastolic function (diastolic class pseudo-normal [class 3] or restrictive [class 4] in 27%) than did those in control (6%) or DM (13%) groups (p = 0.020). Despite these findings, cardiac biomarkers were not significantly different between groups (NTproBNP p = 0.213; cTnI p = 0.138). A summary of echocardiographic findings is presented in Table 2.

**Table 1:** Population characteristics of cats enrolled in the echocardiographic study. Normally distributed data are represented mean (±standard deviation) and compared using a one-way ANOVA. Non-normally distributed data are represented median (range) and compared using a Kruskal-Wallis test. Categorical variables are represented as percentages and compared using a Chi squared test.

Variable	Healthy	Diabetes mellitus	Hypersomatotropism	P value
Number	16	24	67	-
Age (years)	12.4 (10.2–19.4) ^a^	11.5 (5–17)	10.1 (5–15) ^a^	<0.001
Male (%)	69%	79%	66%	0.301
Pedigree (%)	0%	4%	12%	0.381
Body weight (kg)	4.55 (2.68–6.46) ^a^	4.48 (2.84–7.40) ^b^	5.70 (2.0–10.5) ^a^^,^^b^	0.003
Body condition score ≥6/9 ^Φ^	28%	37%	24%	0.406
**Clinical data**				
Rectal temperature (°C)	38.1 (±0.3)	38.1 (±0.7)	38.0 (±0.6)	0.651
Respiratory rate (breaths per minute)	36 (20–72)	32 (20–52)	32 (20–80)	0.366
Heart rate (beats/minute)	179 (±18)	178 (±24)	172 (±24)	0.290
Systolic blood pressure (mmHg)	133 (120–169)	156 (105–171)	148 (120–171)	0.870
Murmur present (%)	31%	21%	40%	0.956
Gallop sound present (%)	0%	8%	6%	0.399
Arrhythmia present (%)	0%	4%	3%	0.644
**Laboratory data**				
IGF-1 (ng/ml)	396 (226–728) ^a^	504 (171–749) ^b^	1800 (1001->2000) ^a^^,^^b^	<0.001
Insulin dose (IU per 24h)	.	6 (3–8) ^a^	16 (2–80) ^a^	<0.001
Fructosamine (μmol/L)	.	453 (278–746)	525 (169–1,076)	0.047
NTproBNP (pmol/L)	86 (24–1358)	50 (24–186)	74 (24–1219)	0.213
Cardiac troponin I (ng/ml)	0.02 (0–0.22)	0.04 (0.02–0.1)	0.03 (0.01–0.23)	0.138

*IGF-1*, *insulin-like growth factor 1*;

^a/b^ indicates a statistically significant difference when pairwise comparison between these factors was performed

^Φ^ Laflamme D. Development and validation of a body condition score system for cats: a clinical tool. 1997 Feline Practice **25** 13 e18

**Table 2:** Echocardiographic measurements of all cats enrolled in the study. Normally distributed data are represented mean (±standard deviation) and compared using a one-way ANOVA. Non-normally distributed data are represented median (range) and compared using a Kruskal-Wallis test. Categorical variables are represented as percentages and compared using a Chi squared test.

Variable	Healthy	Diabetes mellitus	Hypersomatotropism	P value
IVSd (mm)	4.9 (3.9–6.5) ^a^	5.7 (4.2–9.1) ^b^	6.5 (4.1–10.1) ^a^^,^^b^	<0.001
LVFWd (mm)	4.6 (3.6–6.2) ^a^	5.3 (3.8–7.3) ^b^	5.9 (4.1–10.0) ^a^^,^^b^	<0.001
Maximum LV thickness (mm)	5.2 (4.1–6.5) ^a^	5.9 (4.2–9.1) ^b^	6.5 (4.4–10.1) ^a^^,^^b^	<0.001
LVIDd (mm)	14.2 (11.2–17.6) ^a^	15.4 (11.2–20.3)	16.1 (11.9–20.8) ^a^	0.013
LV FS (%)	51 (±7.6)	45 (±7.8)	46 (±3.4)	0.351
LA diameter (mm)	14.0 (12.6–17.4) ^a^	15.2 (10.1–21.3)	16.6 (13.0–29.5)^a^	<0.001
LA:Ao ratio	1.3 (1.0–2.2) ^a^	1.3 (1.0–2.0)	1.6 (1.0–2.9) ^a^	<0.001
LA FS (%)	31 (20–50)	30 (14–47)	29 (1–63)	0.570
LA appendage flow velocity (msec^-1^)	0.47 (±0.15)	0.41 (±0.12)	0.42 (±0.42)	0.736
LVOT velocity (msec^-1^)	1.0 (±0.3)	0.94 (±0.2) ^a^	1.2 (±0.3) ^a^	0.008
RVOT velocity (msec^-1^)	0.9 (±0.3)	1.0 (±0.4)	0.9 (±0.2)	0.911
Mitral E velocity (msec^-1^)	0.7 (±0.12)	0.7 (±0.19)	0.8 (±0.17)	0.275
E deceleration time (ms)	77 (±18)	74 (±13)	74 (±23)	0.758
Mitral A velocity (msec^-1^)	0.6 (±0.17)	0.6 (±0.12)	0.6 (±0.18)	0.450
Mitral E:A ratio	1.2 (0.7–1.9)	1.2 (0.8–2.0)	1.2 (0.8–4.8)	0.296
Diastolic class ≥3	6% ^a^	13%	27% ^a^	0.020
TDI S’s velocity (msec^-1^)	0.10 (±0.03)	0.09 (±0.02)	0.09 (±0.03)	0.297
TDI E’s velocity (msec^-1^)	0.09 (±0.02) ^a^^,^^b^	0.06 (±0.02) ^a^	0.07 (±0.02) ^b^	0.004
TDI A’s velocity (msec^-1^)	0.07 (±0.03)	0.09 (±0.03)	0.08 (±0.03)	0.476
Mitral E: TDI E’ ratio	9.0 (±1.7)	10.6 (±2.5)	12.3 (±4.3)	0.027
Aortic insufficiency (%)	0% ^a^^,b^	17% ^a^	31% ^b^	0.006
Systolic anterior motion of the mitral valve	12%	4%	9%	0.592

^a/b^ indicates a statistically significant difference when pairwise comparison between these factors was performed

### Changes in echocardiographic variables after treatment of hypersomatotropism

In the HS group, clinical and echocardiographic data were available at a minimum of 6 months after treatment for 9 cats that received long-acting pasireotide medical treatment (PAS group) and 20 cats that underwent surgical hypophysectomy (HYP group). There were no statistically significant differences between these groups at baseline. Median follow-up time for treated cats was 6 months (range 6–24 months) in the HYP group and 12 months (range 6–24 months) in the PAS group. A statistically significant reduction in all measures of diastolic LV wall thickness and LA size was present in the HYP group, whereas only reduction in LA diameter was present in the PAS cats (Table 3). Prior to treatment, 65% of cats in the hypophysectomy group had clinically significant LV hypertrophy (≥6mm); after treatment, this reduced to 15%. There was no change in the proportion of cats affected by aortic insufficiency after either treatment, but HYP cats showed an improvement in diastolic function, with fewer cats having pseudonormal or restrictive function (p = 0.013).

**Table 3:** Echocardiographic and plasma IGF-1 measurements before and after treatment of acromegaly.

Echocardiographic parameter	Pre-treatment	Post-treatment	P value
**Hypophysectomy group (n = 20)**			
IVSd (mm)	5.9 (4.4–8.1)	4.9 (3.6–6.6)	<0.001 *
LVFWd (mm)	5.5 (4.2–8.9)	4.9 (3.7–8.4)	0.001 *
LVmax (mm)	6.1 (4.4–8.9)	5.2 (3.9–8.4)	0.002 *
LA diameter (mm)	16.3 (13.0–21.0)	14 (11.5–16.6)	0.001 *
LVIDd (mm)	16.8 (11.9–19.2)	15.1 (10.2–19.4)	0.01 *
LV FS (%)	43 (20–69)	51 (36–74)	0.117
LA FS (%)	28 (15–57)	31 (18–58)	0.627
IGF-1 (ng/mL)	1961 (1055–2000)	85 (15–721)	0.008 *
**Pasireotide group (n = 9)**			
IVSd (mm)	6.7 (5.5–8.0)	6.1 (4.8–8.0)	0.575
LVFWd (mm)	6.2 (5.8–8.2)	5.8 (4.4–7.5)	0.208
LVmax (mm)	6.7 (5.9–8.2)	6.1 (5–8)	0.575
LA diameter (mm)	17.4 (14.1–22.4)	15.6 (11.8–19.3)	0.028 *
LVIDd (mm)	15.5 (12.6–17.7)	15.9 (11.3–17.9)	1.000
LV FS (%)	45 (41–60)	47 (29–51)	0.161
LA FS (%)	34 (27–60)	31 (22–42)	0.465
IGF-1 (ng/mL)	2000 (993–2000)	1659 (18–2000)	0.080

*indicates statistical significance at the 5% level

### Evaluation of myocardial tissue samples

Twenty-one cats suitable for inclusion in the HS group were selected; therefore 21 cases were also selected for each of the HCM and control groups. Pituitary histopathology was available for review in 16/21 cats with acromegaly. Findings were consistent with acidophil adenoma in 11 (69%) and acidophil hyperplasia in 5 (31%). Of the remaining cases where tissue was not available for review, reports were all consistent with either of these diagnoses. None of the 21 cats in the HS group had tissue findings consistent with acidophil carcinoma. Population characteristics and gross pathology findings of all cats are shown in Table 4. Absolute heart weight was significantly greater in cats with HCM (p<0.001) and cats with HS (p = 0.004) than the control group. There was no significant difference in relative heart weight between groups. There was no significant difference in ventricular wall thickness between groups following pairwise comparisons with Bonferroni correction for IVS and LVFW thickness measurements.

**Table 4:** Population characteristics of cats whose myocardial tissue was analysed in the histopathologic study. *HCM*, *hypertrophic cardiomyopathy; HW*, *heart weight; BW*, *body weight; RVFW*, *right ventricular free wall thickness; IVS*, *interventricular septal thickness; LVFW*, *left ventricular wall thickness*.

	Control	HCM	Hypersomatotropism	p-value
Number	21	21	21	-
Male (%)	38	71	57	0.080
Pedigree (%)	43	14	10	0.030
Age (years)	4.0 (2.0–10.0) ^a^^,^^b^	8.0 (3.0–15.0) ^a^	10.8 (3.1–14.6) ^b^	<0.001
Body weight (kg)	4.2 (2.0–8.0)	4.9 (3.9–7.0)	5.4 (3.1–8.3)	0.024
Absolute heart weight (g)	15.5 (10.8–32.6) ^a^^,^^b^	27.4 (18.0–58.0) ^a^	25.0 (19.7–37.6) ^b^	<0.001
HW: BW (%)	0.39 (0.29–0.68)	0.63 (0.26–1.4)	0.47 (0.38–0.60)	0.160
RVFW (mm)	1.9 (0.9–2.9)	2.5 (1.0–4.0)	2.0 (1.0–5.0)	0.096
IVS (mm)	7.0 (3.0–9.0)	9.5 (4.0–12.0)	8.0 (4.0–12.0)	0.032
LVFW (mm)	8.0 (3.0–12.0)	10.0 (3.0–18.0)	8.0 (4.0–12.0)	0.024

^a/b^ indicates a statistically significant difference when pairwise comparison between these factors was performed

**Fig 4:**
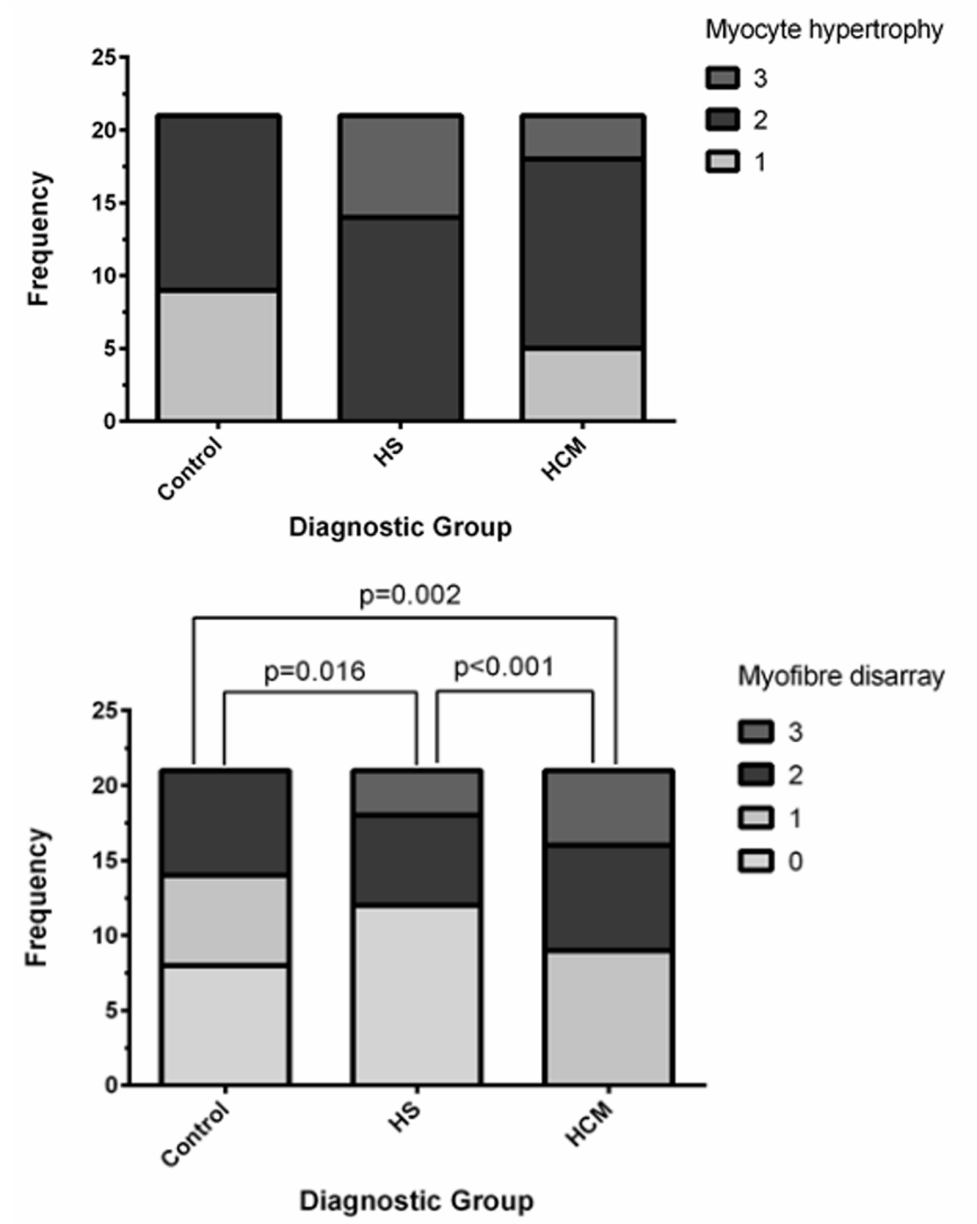
Box plot to show the proportion of cats in each group affected by different grades of (top) myocyte hypertrophy and (bottom) myocyte disarray. No cats reviewed were classified as non-hypertrophic.

**Fig 5:**
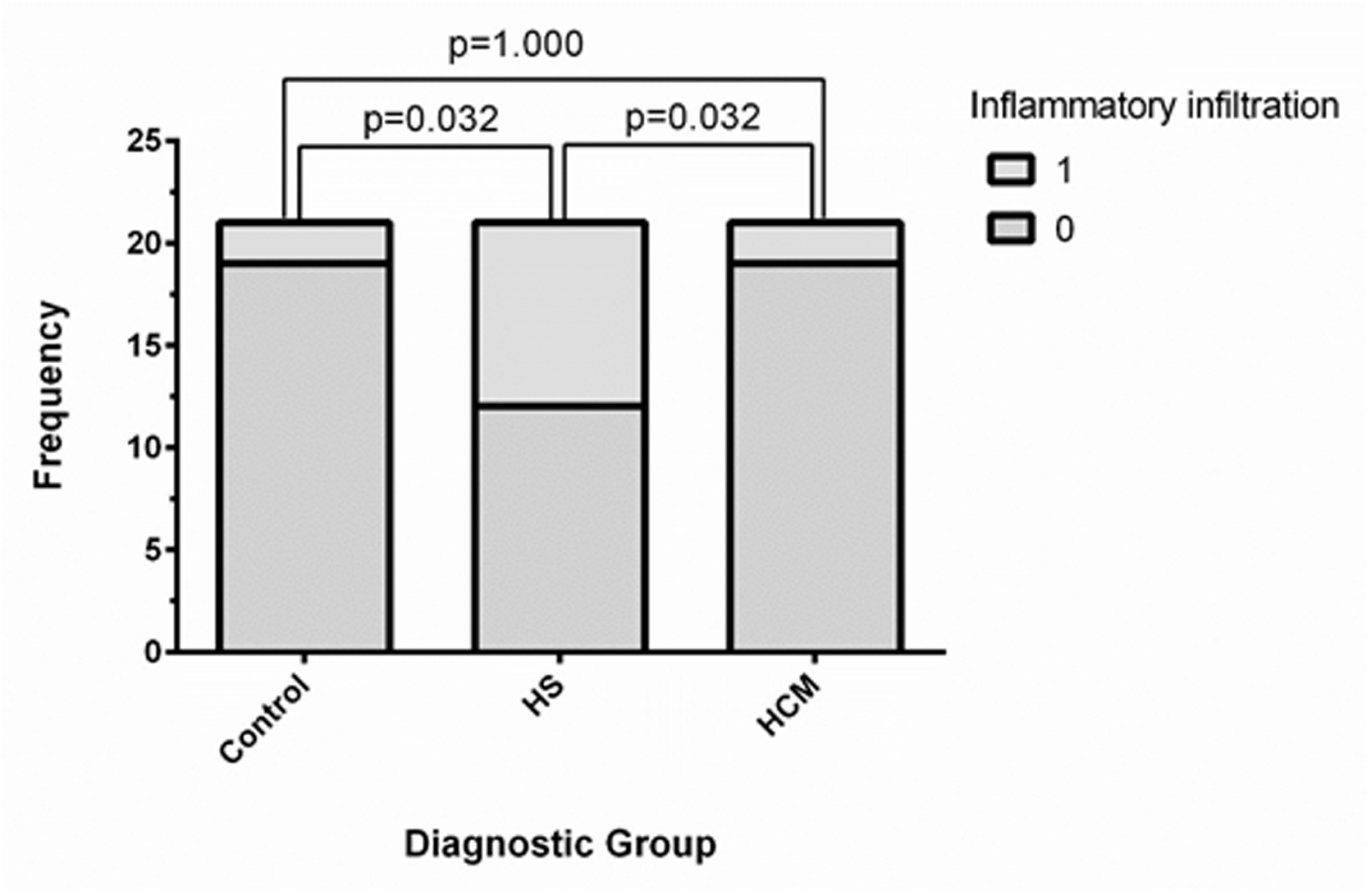
Box plot to show the proportion of cats affected by myocardial inflammatory cell infiltrate in each group.

Myocyte hypertrophy was present in all 63 cats reviewed. In the control group, no cat had severe myocyte hypertrophy, whereas severe hypertrophy was detected in some cats of both the HCM and HS groups (Fig 4). There was no significant difference between the groups in quantitative scores for perivascular fibrosis (p = 0.249), subendocardial fibrosis (p = 0.092), replacement fibrosis (p = 0.672) or intramural arteriosclerosis (p = 0.364). Interstitial fibrosis was more frequent in the HS group than the control group (p<0.001). There was no significant difference in interstitial fibrosis between the HS and HCM (p = 0.343) or HCM and control (p = 0.052) groups. Myofiber disarray was more frequently observed in the HCM group than the HS (p<0.001) or control (p = 0.002) groups, and in the control group than the HS group (p = 0.016). There was a significant difference in inflammatory cell infiltration (p = 0.017, Fig 5) and myocyte degeneration (0.037) between groups, however no significant difference remained following pair-wise analysis.

## Discussion

These data strongly suggest that hypersomatotropism in cats is associated with left ventricular hypertrophy, which is largely reversible following hypophysectomy. The clinical, echocardiographic and histopathological findings described in this study closely mimic those detected in humans with acromegalic cardiomyopathy. Therefore, we propose that feline acromegalic cardiomyopathy represents an attractive spontaneously occurring model of the human disease, and its study could bridge the gap between engineered rodent models and the human condition. The relatively high prevalence of the disease in the cat, compared to humans, further offers an attractive characteristic for its future study.

This is the first study to deliver a detailed description of feline acromegalic cardiomyopathy and to offer comparison with non-acromagelic diabetic and apparently healthy control cats. Feline acromegalic cardiomyopathy is characterised *ante mortem* by echocardiographic evidence of LV hypertrophy and diastolic dysfunction together with an increased prevalence of aortic insufficiency and left atrial dilation (presumably secondary to LV diastolic dysfunction and volume loading effects of diabetes) compared to control cats. These findings support those reported in previous case series [28, 37], but this time on the basis of rigorous comparison of cats with acromegaly to a suitable population of controls.

It is interesting to note the relatively limited reverse myocardial remodelling of cats treated with pasireotide compared to hypophysectomy. This might be explained by the fact that IGF-1 did not reduce significantly in those cats treated medically, in contrast to hypophysectomy-treated cats. In one recent case series, a statistically significant reduction in IGF-1 was reported after treatment with pasireotide, but the magnitude of change reported was much lower than for the cats undergoing hypophysectomy in this study [46]. Without further study in a larger cohort of cats using a treat-to-target protocol, it is impossible to say whether or not long-term medical management of acromegalic cardiomyopathy with somatostatin receptor agonists is inferior to surgical treatment. Interestingly, both medical and surgical treatment of HS in humans is associated with a reduction in LV mass [24]. In humans, older patients with a longer duration of HS have more severe cardiac dysfunction and a less profound treatment response than younger patients [16, 53–64]. These older patients are also more likely to be diabetic [65]. Our population of cats, all of whom were diabetic, may represent a longer duration of disease and therefore the response to medical treatment may be less profound than seen in most humans with acromegaly. The dose used in this study may have been inadequate, or a species difference in response to somatostatin analogs between cats and humans is possible.

Similarities between the cardiac phenotype in cats with HS and human acromegalic cardiomyopathy were also observed at the tissue level. In humans, myocyte hypertrophy was present in 93% patients (100% cats), interstitial fibrosis in 85% (>90% cats), intramural arteriosclerosis in 22% (approximately 50% cats) and inflammatory cell infiltrates in 59% (approximately 40% cats). Similarly to cats, myocyte disarray is uncommon [25]. In humans, reversal of histopathological abnormalities has been observed after treatment with octreotide [64]. It was not possible to obtain myocardial samples post-hypophysectomy in this study due to excellent long-term survival rates. Myocardial biopsy is not routinely performed in cats, so assessment of myocardial changes pre- and post-treatment in the same cats was impossible.

Evidence of myocardial hypertrophy was common in all groups of cats studied, both echocardiographically and histopathologically. Clinically significant LV hypertrophy (>6mm) was identified in 21% of control cats using echocardiography and histopathological evidence of myocyte hypertrophy was present in 100% of control cats at post-mortem. Echocardiographic prevalence of HCM has been reported at 29.4% in a population of apparently healthy cats >9 years old [40], and one recent histopathological study [66] found that quantitative measures of myofiber diameter were not significantly different between hearts from cats with HCM and controls, even though myocyte hypertrophy is considered one of the principal changes in feline HCM [1–4]. There is no published reference for feline myocyte size, and therefore many studies (including this one) use a semiquantitative scoring system. The main problem with using such a system is that there is little room to account for normal biologic variation. It is possible that the “mild-focal” hypertrophy reported in the control group represented a normal variant in older cats. Clearly, a more robust reference for myocyte size is required in cats to facilitate greater accuracy in future research.

As expected, myofiber disarray—considered the histopathologic gold standard for diagnosis of HCM [2, 67, 68]—was more frequently observed in the HCM group than the HS or control groups. Myofiber disarray was also found more frequently in the control group than the HS group. This is most likely explained by a random difference in prevalence of HCM between groups, as both groups may have included cats with concurrent (incidental) HCM.

Interstitial fibrosis was more frequent in the HS group than the control group, but not the HCM group. Interstitial fibrosis is considered a characteristic histopathological finding in HCM in both humans and cats [1], and has also been described in both human [25] and feline [28] patients with acromegaly. Increased collagen turnover has also been demonstrated both in the cat [69] and human [70] with acromegaly. One previous study in humans found that myocardial fibrosis increases with age [71], so the presence of fibrosis in itself may not always indicate disease. Despite attempting to age-match the groups in our histopathological study, cats in the control group were significantly younger than in the HS and HCM groups. Therefore, it remains possible that interstitial fibrosis was related to age rather than disease. There was no significant difference between groups in the degree of replacement fibrosis or intramural arteriosclerosis. The latter has previously been reported in association with feline HS [28], but it is often also included as a diagnostic criterion for HCM [72]. Once again, the prevalence of incidental cardiomyopathy in geriatric cats may explain the lack of significant differences between groups.

A crucial difference between humans and cats with acromegalic cardiomyopathy is the proportion of individuals affected by DM. All cats with acromegaly in our study had DM, and only four cases of non-diabetic acromegalic cats have been reported in the literature thus far [32–33]. In contrast, DM is only reported in 15–35% of humans with HS [65, 73]. Because of this, determining which myocardial changes are caused by the diabetic state and which directly relate to over-secretion of GH and IGF-1 is a challenge in cats. In this study, the non-acromegalic diabetic state (DM group) did not appear to be associated with clinically significant myocardial hypertrophy. However, the group size was relatively small and a subtle effect of diabetes may not have been detected. Previously, diabetic cats have been reported to exhibit a progressive diastolic dysfunction, but LV hypertrophy was not identified in these cats [19]. Additionally, in the present study, histopathology on non-acromegalic diabetic cats was not included in the statistical analysis due to lack of suitable material. Despite this, our data suggests that the GH and IGF-1 excess of acromegaly has significant effects on the myocardium over and above any changes induced by diabetes alone. Despite the relatively rapid reversal of these changes when the acromegaly was treated effectively through surgery, we cannot be entirely certain from this study that DM itself was not the cause of the reversible changes. We would recommend that a larger cohort of diabetic cats is studied, with suitable control population for comparison, before the possibility of a diabetic cardiomyopathy in cats is excluded. What we can say from this data is that the feline acromegalic cardiomyopathy, albeit one associated with concurrent diabetes in this population, was largely reversible with treatment by hypophysectomy, excluding the possibility that hypertrophy in the HS group was caused by primary myocardial disease.

In summary, this report describes in detail the cardiac manifestations of spontaneously occurring feline hypersomatotropism at the clinical and histopathological level. Many phenotypic similarities with human acromegalic cardiomyopathy were identified. Furthermore, we have refined the phenotype of this model by comparing cats with acromegaly cats to those with potential confounding diseases, such as HCM and diabetes. We therefore suggest that feline acromegalic cardiomyopathy represents an appealing, naturally occurring model to study, with significant potential to form the foundations of future research. Study of privately-owned pet cats with acromegaly is a realistic option, if owners consent to enrolment of their cat in clinical trials; a research opportunity in a shared environment, with higher disease prevalence, a shorter lifespan and a higher event-rate than humans. Research centred on the naturally-occurring feline disease could facilitate identification of novel therapeutics that may be rapidly translated into the human clinic. Furthermore, the easier acquisition of suitable myocardial tissue from cats (given their shorter lifespan) compared to human patients will facilitate exploration of the cellular pathways which trigger ventricular remodelling; this may allow the development of anti-hypertrophic therapies potentially relevant to other triggers for hypertrophy.

## Supporting information

S1 Data Database 1 baseline. (XLSX)

S2 Data Database 2 treatment. (SAV)

## References

[1] Fox PR, Liu SK & Maron BJ 1995 Echocardiographic assessment of spontaneously occurring feline hypertrophic cardiomyopathy: an animal model of human disease. *Circulation* 92 2645–2651. 7586368 10.1161/01.cir.92.9.2645

[2] Kittleson MD, Meurs KM, Munro MJ, Kittleson JA, Liu S-K, Pion PD & Towbin JA 1999 Familial Hypertrophic Cardiomyopathy in Maine Coon Cats: an animal model of human disease. *Circulation* 99 3172–3180. 10377082 10.1161/01.cir.99.24.3172

[3] Fox PR, Maron BJ, Basso C, Liu SK & Thiene G 2000 Spontaneously Occurring Arrhythmogenic Right Ventricular Cardiomyopathy in the Domestic Cat: A New Animal Model Similar to the Human Disease. *Circulation* 102 1863–1870 11023944 10.1161/01.cir.102.15.1863

[4] Fox PR, Basso C, Thiene G & Maron BJ 2014 Spontaneously occurring restrictive nonhypertrophied cardiomyopathy in domestic cats: a new animal model of human disease. *Cardiovascular Pathology* 23 28–34. doi: 10.1016/j.carpath.2013.08.001 24035181 10.1016/j.carpath.2013.08.001

[5] Peterson ME, Kintzer PP, Cavanagh PG, Fox PR, Ferguson DC, Johnson GF & Becker DJ 1983 Feline hyperthyroidism: pretreatment clinical and laboratory evaluation of 131 cases. *Journal of the American Veterinary Medical Association* 183 103–110. 6874510

[6] Liu SK, Peterson ME & Fox PR 1984 Hypertrophic cardiomyopathy and hyperthyroidism in the cat. *Journal of the American Veterinary Medical Association* 185 52–7. 6540256

[7] Jacobs G, Hutson C, Dougherty J & Kirmayer A 1986 Congestive heart failure associated with hyperthyroidism in cats. *Journal of the American Veterinary Medical Association* 188 52–56. 3944009

[8] Moise NS & Dietze AE 1986 Echocardiographic, electrocardiographic, and radiographic detection of cardiomegaly in hyperthyroid cats. *American Journal of Veterinary Research* 47 1487–1494. 2943199

[9] Bond BR, Fox PR, Peterson ME & Skavaril RV 1988 Echocardiographic findings in 103 cats with hyperthyroidism. *Journal of the American Veterinary Medical Association* 192 1546–1549. 2970449

[10] Fox PR, Peterson ME & Broussard JD 1999 Electrocardiographic and radiographic changes in cats with hyperthyroidism: comparison of populations evaluated during 1992–1993 vs. 1979–1982. *Journal of the American Animal Hospital Association* 35 27–31. doi: 10.5326/15473317-35-1-27 9934924 10.5326/15473317-35-1-27

[11] Connolly DJ, Guitian J, Boswood A & Neiger R 2005 Serum troponin I levels in hyperthyroid cats before and after treatment with radioactive iodine. *Journal of Feline Medicine and Surgery* 7 289–300. doi: 10.1016/j.jfms.2005.01.002 16182184 10.1016/j.jfms.2005.01.002 PMC10822355

[12] Weichselbaum RC, Feeney DA & Jessen CR 2005 Relationship between selected echocardiographic variables before and after radioiodine treatment in 91 hyperthyroid cats. *Veterinary Radiology & Ultrasound* 46 506–513. 16396269 10.1111/j.1740-8261.2005.00099.x

[13] Menaut P, Connolly DJ, Volk A, Pace C, Luis Fuentes V, Elliott J & Syme H 2012 Circulating natriuretic peptide concentrations in hyperthyroid cats. *Journal of Small Animal Practice* 53 673–678. doi: 10.1111/j.1748-5827.2012.01301.x 23146093 10.1111/j.1748-5827.2012.01301.x

[14] Sangster JK, Panciera DL, Abbott JA, Zimmerman KC & Lantis AC 2014 Cardiac Biomarkers in Hyperthyroid Cats. *Journal of Veterinary Internal Medicine* 28 465–472. doi: 10.1111/jvim.12259 24350989 10.1111/jvim.12259 PMC4857992

[15] Dekkers OM, Biermasz NR, Pereira AM, Romijn JA & Vandenbroucke JP 2008 Mortality in acromegaly: a metaanalysis. *The Journal of Clinical Endocrinology and Metabolism* 93 61–67. doi: 10.1210/jc.2007-1191 17971431 10.1210/jc.2007-1191

[16] Colao A, Pivonello R, Grasso LF, Auriemma RS, Galdiero M, Savastano S & Lombardi G 2011 Determinants of cardiac disease in newly diagnosed patients with acromegaly: results of a 10 year survey study. *European Journal of Endocrinology* 165 713–721. doi: 10.1530/EJE-11-0408 21868601 10.1530/EJE-11-0408

[17] Dal J, Feldt-Rasmussen U, Andersen M, Kristensen L, Laurberg P, Pedersen L, Dekkers O, Sørensen H & Jørgensen J 2016 Acromegaly incidence, prevalence, complications and long-term prognosis: a nationwide cohort study. *European Journal of Endocrinology* 175 181–190. doi: 10.1530/EJE-16-0117 27280374 10.1530/EJE-16-0117

[18] Colao A, Ferone D & Marzullo P 2004 Systemic complications of acromegaly: epidemiology, pathogenesis, and management. *Endocrine Reviews* 25 102–152 doi: 10.1210/er.2002-0022 14769829 10.1210/er.2002-0022

[19] Pereira AM, Thiel SW van, Lindner JR, Roelfsema F, Wall EE van der, Morreau H, Smit JW, Romijn JA & Bax JJ 2004 Increased prevalence of regurgitant valvular heart disease in acromegaly. *The Journal of Clinical Endocrinology and Metabolism* 89 71–75. doi: 10.1210/jc.2003-030849 14715829 10.1210/jc.2003-030849

[20] McGuffin WL, Sherman BM, Roth JESSE, Gorden P, Kahn CR, Roberts WC & Frommer PL 1974 Acromegaly and Cardiovascular Disorders. *Annals of Internal Medicine* 81 11–18. 4276173 10.7326/0003-4819-81-1-11

[21] Fazio S, Cittadini A, Sabatini D, Merola B, Colao AM, Biondi B, Lombardi G & Saccá L 1993 Evidence for biventricular involvement in acromegaly: a Doppler echocardiographic study. *European Heart Journal* 14 26–33. 10.1093/eurheartj/14.1.26 8432287

[22] Fazio S, Cittadini A, Cuocolo A, Merola B, Sabatini D, Colao AM, Biondi B, Lombardi G & Saccà L 1994 Impaired cardiac performance is a distinct feature of uncomplicated acromegaly. *Journal of Clinical Endocrinology & Metabolism* 79 441–446. 8045960 10.1210/jcem.79.2.8045960

[23] Fazio S, Sabatini D, Cittadini A, Cocozza M, Cuocolo A, Merola B, Colao A, Biondi B, Lombardi G & Saccà L 1996 Cardiac involvement in active uncomplicated acromegaly. *International Journal of Angiology* 5 55–58.

[24] Colao A, Baldelli R, Marzullo P, Ferretti E, Ferone D, Gargiulo P, Petretta M, Tamburrano G, Lombardi G & Liuzzi A 2000a Systemic hypertension and impaired glucose tolerance are independently correlated to the severity of the acromegalic cardiomyopathy. *The Journal of Clinical Endocrinology and Metabolism* 85 193–199. doi: 10.1210/jcem.85.1.6318 10634386 10.1210/jcem.85.1.6318

[25] Lie JT & Grossman SJ 1980 Pathology of the heart in acromegaly: anatomic findings in 27 autopsied patients. *American Heart Journal* 100 41–52. 6446234 10.1016/0002-8703(80)90277-x

[26] Niessen SJM, Petrie G, Gaudiano F, Khalid M, Smyth JBA, Mahoney P & Church DB 2007 Feline Acromegaly: An Underdiagnosed Endocrinopathy? *Journal of Veterinary Internal Medicine* 21 899–905. 17939541 10.1892/0891-6640(2007)21[899:faaue]2.0.co;2

[27] Niessen S, Forcada Y, Mantis P, Lamb CR, Harrington N, Fowkes R, Korbonits M, Smith K & Church DB 2015 Studying Cat (Felis catus) Diabetes: Beware of the Acromegalic Imposter. *PloS One* e0127794 doi: 10.1371/journal.pone.0127794 26023776 10.1371/journal.pone.0127794 PMC4449218

[28] Peterson ME, Taylor RS, Greco DS, Nelson RW, Randolph JF, Foodman MS, Moroff SD, Morison SA, Lothrop CD 1990 Acromegaly in 14 cats. *Journal of Veterinary Internal Medicine* 4 192–201. 2401966 10.1111/j.1939-1676.1990.tb00897.x

[29] Niessen SJM 2010 Feline Acromegaly An essential differential diagnosis for the difficult diabetic. *Journal of Feline Medicine & Surgery* 12 15–23. 20123483 10.1016/j.jfms.2009.12.003 PMC10845472

[30] Niessen SJM, Church DB & Forcada Y 2013 Hypersomatotropism, acromegaly and hyperadrenocorticism and feline diabetes mellitus. *The Veterinary Clinics of North America*. *Small Animal Practice* 43 319–350. doi: 10.1016/j.cvsm.2012.12.004 23522175 10.1016/j.cvsm.2012.12.004

[31] Rosca M, Forcada Y, Solcan G, Church DB & Niessen SJM 2013 Screening diabetic cats for hypersomatotropism: performance of an enzyme-linked immunosorbent assay for insulin-like growth factor 1. *Journal of Feline Medicine and Surgery* 16 82–88. doi: 10.1177/1098612X13496246 23828811 10.1177/1098612X13496246 PMC11383139

[32] Fletcher JM, Scudder CJ, Kiupel M, Pipe-Martin HN, Kenny PJ, Mantis P, Fenn J, Smith K, Blair RV, Granger LA et al 2016 Hypersomatotropism in 3 Cats without Concurrent Diabetes Mellitus. *Journal of Veterinary Internal Medicine* 30 1216–1221. doi: 10.1111/jvim.14360 27255700 10.1111/jvim.14360 PMC5089606

[33] Fracassi F, Salsi M, Sammartano F, Bo S & Kooistra HS 2016 Acromegaly in a non-diabetic cat. *Journal of Feline Medicine and Surgery Open Reports* doi: 10.1177/2055116916646585 28491423 10.1177/2055116916646585 PMC5362850

[34] Dirtu AC, Niessen SJ, Jorens PG & Covaci A 2013 Organohalogenated contaminants in domestic cats’ plasma in relation to spontaneous acromegaly and type 2 diabetes mellitus: a clue for endocrine disruption in humans? *Environment International* 57–58 60–67. doi: 10.1016/j.envint.2013.04.004 23672960 10.1016/j.envint.2013.04.004

[35] Posch B, Dobson J & Herrtage M 2011 Magnetic resonance imaging findings in 15 acromegalic cats. *Veterinary Radiology & Ultrasound* 52 422–427. 21447042 10.1111/j.1740-8261.2011.01821.x

[36] Lamb CR, Ciasca TC, Mantis P, Forcada Y, Potter M, Church DB, Niessen SJ 2014 Computed tomographic signs of acromegaly in 68 diabetic cats with hypersomatotropism. *Journal of Feline Medicine and Surgery* 16 99–108. doi: 10.1177/1098612X13497212 23847300 10.1177/1098612X13497212 PMC11383125

[37] Myers JA, Lunn KF & Bright JM 2014 Echocardiographic findings in 11 cats with acromegaly. *Journal of Veterinary Internal Medicine* 28 1235–1238. doi: 10.1111/jvim.12386 24962737 10.1111/jvim.12386 PMC4857958

[38] Paige C, Abbott J, Elvinger F & Pyle L 2009 Prevalence of cardiomyopathy in apparently healthy cats. *Journal of the American Veterinary Medical Association* 20 1398–1403. 10.2460/javma.234.11.1398 19480619

[39] Wagner T, Luis Fuentes V, Payne JR, McDermott N & Brodbelt D 2010 Comparison of auscultatory and echocardiographic findings in healthy adult cats. *Journal of Veterinary Cardiology* 12 171–182. doi: 10.1016/j.jvc.2010.05.003 21075067 10.1016/j.jvc.2010.05.003

[40] Payne JR, Brodbelt DC & Luis Fuentes V 2015 Cardiomyopathy prevalence in 780 apparently healthy cats in rehoming centres (the CatScan study). *Journal of Veterinary Cardiology* 17 S244–257. doi: 10.1016/j.jvc.2015.03.008 26776583 10.1016/j.jvc.2015.03.008

[41] Galderisi M, Anderson KM, Wilson PWF & Levy D 1991 Echocardiographic evidence for the existence of a distinct diabetic cardiomyopathy (the Framingham Heart Study). *The American Journal of Cardiology* 68 85–89. 2058564 10.1016/0002-9149(91)90716-x

[42] Murarka S & Movahed MR 2010 Diabetic cardiomyopathy. *Journal of Cardiac Failure* 16 971–979. doi: 10.1016/j.cardfail.2010.07.249 21111987 10.1016/j.cardfail.2010.07.249

[43] Little CJL & Gettinby G 2008 Heart failure is common in diabetic cats: findings from a retrospective case‐controlled study in first‐opinion practice. *Journal of Small Animal Practice* 49 17–25. doi: 10.1111/j.1748-5827.2007.00466.x 18173774 10.1111/j.1748-5827.2007.00466.x

[44] Pereira NJ, Novo Matos J, Baron Toaldo J, Bartoszuk U, Summerfield N, Riederer A, Reusch C & Glaus TM 2017 Cats with diabetes mellitus have diastolic dysfunction in the absence of structural heart disease. *The Veterinary Journal* 225 50–55. doi: 10.1016/j.tvjl.2017.04.017 28720299 10.1016/j.tvjl.2017.04.017

[45] Brown S, Atkins C, Bagley R, Carr A, Cowgill L, Davidson M, Egner B, Elliott J, Henik R, Labato M et al 2007 Guidelines for the identification, evaluation, and management of systemic hypertension in dogs and cats. *Journal of Veterinary Internal Medicine* 21 542–58. 17552466 10.1892/0891-6640(2007)21[542:gftiea]2.0.co;2

[46] Gostelow R, Scudder CJ, Keyte SV, Forcada Y, Fowkes RC, Schmid HA, Church DB & Niessen SJM 2017 Pasireotide Long‐Acting Release Treatment for Diabetic Cats with Underlying Hypersomatotropism. *Journal of Veterinary Internal Medicine* 31 355–364. doi: 10.1111/jvim.14662 28145031 10.1111/jvim.14662 PMC5354018

[47] Meij BPP, Auriemma E, Grinwis G, Buijtels JJ & Kooistra HS 2010 Successful treatment of acromegaly in a diabetic cat with transsphenoidal hypophysectomy. *Journal of Feline Medicine and Surgery* 12 406–410. doi: 10.1016/j.jfms.2010.03.014 20417901 10.1016/j.jfms.2010.03.014 PMC11318759

[48] Wilkie LJ, Smith K & Luis Fuentes V 2015 Cardiac pathology findings in 252 cats presented for necropsy; a comparison of cats with unexpected death versus other deaths. *Journal of Veterinary Cardiology* 17 S329–340 doi: 10.1016/j.jvc.2015.09.006 26776590 10.1016/j.jvc.2015.09.006

[49] Rosol T. (2016). Endocrine Glands In: Maxie M *Pathology of Domestic Animals*. 6th ed Missouri: Elsevier 271–272

[50] Moise NS, Dietze AE, Mezza LE, Strickland D, Erb HN & Edwards NJ 1986 Echocardiography, electrocardiography, and radiography of cats with dilatation cardiomyopathy, hypertrophic cardiomyopathy, and hyperthyroidism *American Journal of Veterinary Research* 47 1476–1486. 2943198

[51] Lesser M, Fox PR, Bond BR 1992 Assessment of hypertension in 40 cats with left ventricular hypertrophy by Doppler-shift sphygmomanometry. *Journal of Small Animal Practice* 33 55–58.

[52] Maxie Grant M 2007 The Cardiovascular System Ch2 In Jubb K, Palmer J. *Pathology of Domestic Animals* vol.3 edn 5 pp737 Ed Maxie Grant M. Cambridge; Elsevier Saunders.

[53] Lombardi G, Colao A, Ferone D, Marzullo P, Landi ML, Longobardi S, Iervolino E, Cuocolo A, Fazio S, Merola B et al 1996 Cardiovascular aspects in acromegaly: effects of treatment. *Metabolism*: *Clinical and Experimental* 45 57–60. 10.1016/s0026-0495(96)90083-9 8769383

[54] Lombardi G, Colao A, Marzullo P, Biondi B, Palmieri E & Fazio S 2002 Improvement of left ventricular hypertrophy and arrhythmias after lanreotide-induced GH and IGF-I decrease in acromegaly. A prospective multi-center study. *Journal of Endocrinological Investigation* 25 971–976. doi: 10.1007/BF03344070 12553557 10.1007/BF03344070

[55] Baldelli R, Colao A, Razzore P, Jaffrain-Rea ML, Marzullo P, Ciccarelli E, Ferretti E, Ferone D, Gaia D, Camanni F et al 2000 Two-year follow-up of acromegalic patients treated with slow release lanreotide (30 mg). *The Journal of Clinical Endocrinology and Metabolism* 85 4099–4103. doi: 10.1210/jcem.85.11.6948 11095439 10.1210/jcem.85.11.6948

[56] Colao A, Cuocolo A, Marzullo P, Nicolai E, Ferone D, Florimonte L, Salvatore M & Lombardi G 1999a Effects of 1-year treatment with octreotide on cardiac performance in patients with acromegaly. *The Journal of Clinical Endocrinology and Metabolism* 84 17–23. doi: 10.1210/jcem.84.1.5368 9920056 10.1210/jcem.84.1.5368

[57] Colao A, Marzullo P, Ferone D, Spinelli L, Cuocolo A, Bonaduce D, Salvatore M, Boerlin V, Lancranjan I & Lombardi G 2000b Cardiovascular effects of depot long-acting somatostatin analog Sandostatin LAR in acromegaly. *The Journal of Clinical Endocrinology and Metabolism* 85 3132–3140. doi: 10.1210/jcem.85.9.6782 10999798 10.1210/jcem.85.9.6782

[58] Colao A, Cuocolo A, Marzullo P, Nicolai E, Ferone D, Della Morte AM, Pivonello R, Salvatore M & Lombardi G 2001 Is the acromegalic cardiomyopathy reversible? Effect of 5-year normalization of growth hormone and insulin-like growth factor I levels on cardiac performance. *The Journal of Clinical Endocrinology and Metabolism* 86 1551–1557. doi: 10.1210/jcem.86.4.7376 11297582 10.1210/jcem.86.4.7376

[59] Colao A, Marzullo P & Lombardi G 2002 Effect of a six-month treatment with lanreotide on cardiovascular risk factors and arterial intima-media thickness in patients with acromegaly. *European Journal of Endocrinology* 146 303–309. 11888835 10.1530/eje.0.1460303

[60] Colao A, Auriemma RS, Galdiero M, Lombardi G & Pivonello R 2009 Effects of initial therapy for five years with somatostatin analogs for acromegaly on growth hormone and insulin-like growth factor-I levels, tumor shrinkage, and cardiovascular disease: a prospective study. *The Journal of Clinical Endocrinology and Metabolism* 94 3746–3756. doi: 10.1210/jc.2009-0941 19622615 10.1210/jc.2009-0941

[61] Gilbert J, Ketchen M, Kane P, Mason T, Baister E, Monaghan M, Barr S & Harris PE 2003 The treatment of de novo acromegalic patients with octreotide-LAR: efficacy, tolerability and cardiovascular effects. *Pituitary* 6 11–18. 14674719 10.1023/a:1026273509058

[62] Jaffrain-Rea M-LL, Minniti G, Moroni C, Esposito V, Ferretti E, Santoro A, Infusino T, Tamburrano G, Cantore G & Cassone R 2003 Impact of successful transsphenoidal surgery on cardiovascular risk factors in acromegaly. *European Journal of Endocrinology* 148 193–201. 12590638 10.1530/eje.0.1480193

[63] Pivonello R, Galderisi M, Auriemma RS, Martino MC De, Galdiero M, Ciccarelli A, D’Errico A, Kourides I, Burman P, Lombardi G et al 2007 Treatment with growth hormone receptor antagonist in acromegaly: effect on cardiac structure and performance. *The Journal of Clinical Endocrinology and Metabolism* 92 476–482. doi: 10.1210/jc.2006-1587 17105844 10.1210/jc.2006-1587

[64] Nishiki M, Murakami Y, Sohmiya M, Koshimura K, Inoue K, Goto Y, Nakamura N & Kato Y 1997 Histopathological improvement of acromegalic cardiomyopathy by intermittent subcutaneous infusion of octreotide. *Endocrine Journal* 44 655–660. 9466320 10.1507/endocrj.44.655

[65] Fieffe S, Morange I, Petrossians P, Chanson P, Rohmer V, Cortet C, Borson-Chazot F, Brue T & Delemer B 2011 Diabetes in acromegaly, prevalence, risk factors, and evolution: data from the French Acromegaly Registry. *European Journal of Endocrinology* 164 877–884. doi: 10.1530/EJE-10-1050 21464140 10.1530/EJE-10-1050

[66] Kershaw O, Heblinski N, Lotz F, Dirsch O & Gruber AD 2012 Diagnostic Value of Morphometry in Feline Hypertrophic Cardiomyopathy. *Journal of Comparative Pathology* 147 73–83. doi: 10.1016/j.jcpa.2011.11.196 22297077 10.1016/j.jcpa.2011.11.196

[67] Liu SK, Tilley LP & Lord PF 1975 Feline cardiomyopathy. *Recent Advances in Studies on Cardiac Structure and Metabolism* 10 627–40. 128793

[68] Fox PR 2003 Hypertrophic Cardiomyopathy. Clinical and Pathologic Correlates. *Journal of Veterinary Cardiology* 5 39–45. 10.1016/S1760-2734(06)70051-0 19081364

[69] Keyte SV, Kenny PJ, Forcada Y, Church DB & Niessen SJM 2016 Serum N‐Terminal Type III Procollagen Propeptide: An Indicator of Growth Hormone Excess and Response to Treatment in Feline Hypersomatotropism. *Journal of Veterinary Internal Medicine* 30 973–982. doi: 10.1111/jvim.14373 27425382 10.1111/jvim.14373 PMC5108467

[70] Piovesan A, Terzolo M, Reimondo G, Pia A, Codegone A, Osella G, Boccuzzi A, Paccotti P & Angeli A 1994 Biochemical markers of bone and collagen turnover in acromegaly or Cushing’s syndrome. *Hormone and Metabolic Research* 26 234–237. doi: 10.1055/s-2007-1001672 8076906 10.1055/s-2007-1001672

[71] Liu C-Y, Liu Y-C, Wu C, Armstrong A, Volpe G, Geest R, Liu Y, Hundley W, Gomes A, Liu S et al 2013 Evaluation of Age-Related Interstitial Myocardial Fibrosis With Cardiac Magnetic Resonance Contrast-Enhanced T1 Mapping: MESA (Multi-Ethnic Study of Atherosclerosis). *Journal of the American College of Cardiology* 62 1280–1287. doi: 10.1016/j.jacc.2013.05.078 23871886 10.1016/j.jacc.2013.05.078 PMC3807823

[72] Maron BJ & Fox PR 2015 Hypertrophic cardiomyopathy in man and cats. *Journal of Veterinary Cardiology* 17 S6–S9. doi: 10.1016/j.jvc.2015.03.007 26776595 10.1016/j.jvc.2015.03.007

[73] Mestron A, Webb SM, Astorga R, Benito P, Catala M, Gaztambide S, Gomez J-MM, Halperin I, Lucas-Morante T, Moreno B et al 2004 Epidemiology, clinical characteristics, outcome, morbidity and mortality in acromegaly based on the Spanish Acromegaly Registry (Registro Espanol de Acromegalia, REA). *European Journal of Endocrinology* 151 439–446. 15476442 10.1530/eje.0.1510439

